# Birth weight variants are associated with variable fetal intrauterine growth from 20 weeks of gestation

**DOI:** 10.1038/s41598-018-26752-3

**Published:** 2018-05-30

**Authors:** L. Engelbrechtsen, D. Gybel-Brask, Y. Mahendran, M. Crusell, T. H. Hansen, T. M. Schnurr, E. Hogdall, L. Skibsted, T. Hansen, H. Vestergaard

**Affiliations:** 1 Novo Nordisk Foundation Center for Basic Metabolic Research, Section of Metabolic Genetics, University of Copenhagen, Copenhagen, Denmark; 2 Danish Diabetes Academy, Odense, Denmark; 3 Department of Gynecology and Obstetrics, Section of Fetal Medicine, Roskilde University Hospital, Roskilde, Denmark; 4 Molecular Unit, Department of Pathology, Herlev Hospital, University of Copenhagen, Herlev, Denmark; 5 Steno Diabetes Center Copenhagen, Gentofte, Denmark

## Abstract

Fetal intrauterine growth is influenced by complex interactions between the maternal genes, environment and fetal genes. The aim of this study was to assess the effect of GWAS-identified genetic variants associated with birth weight on intrauterine fetal growth in 665 children. Fetal growth was estimated by two-dimensional ultrasound scans at 20, 25 and 32 weeks of gestation and growth trajectories were modeled using mixed linear regression. A genetic risk score (GRS) of birth weight-raising variants was associated with intrauterine growth showing an attenuating effect on the unconditional daily reduction in proportional weight gain of 8.92 × 10^−6^ percentage points/allele/day (p = 2.0 × 10^−4^), corresponding to a mean difference of 410 g at 40 weeks of gestation between a child with lowest and highest GRS. Eight variants were independently associated with intrauterine growth throughout the pregnancy, while four variants were associated with fetal growth in the periods 20–25 or 25–32 weeks of gestation, indicating that some variants may act in specific time windows during pregnancy. Four of the intrauterine growth variants were associated with type 2 diabetes, hypertension or BMI in the UK Biobank, which may provide basis for further understanding of the link between intrauterine growth and later risk of metabolic disease.

## Introduction

Fetal intrauterine growth is influenced by complex interactions between the maternal genes and environment, and fetal genes^1^. Maternal factors, such as obesity and diabetes, can result in abnormal fetal growth patterns leading to large-for-gestational age (LGA) fetuses, whereas acquired complications during pregnancy such as pre-eclampsia can lead to small-for-gestational age (SGA) fetuses^1–3^. Abnormal fetal growth can increase the risk of perinatal adverse effects, but also the risk of morbidity and mortality in adult life^4–6^.

An association between fetal growth and the occurrence of metabolic syndrome later in life was first noted by Barker *et al*. in 1993^7^. This lead to a number of observational studies, which demonstrated that adverse environmental factors during fetal development increase the lifelong risk of metabolic and cardiovascular complications^1,2,5–12^. Subsequently, the maternal factors associated with birth weight have been carefully studied in numerous epidemiological studies^2,8,10,12^.

Knowledge about the fetal genetic contribution to intrauterine growth is limited. A recent genome-wide association study (GWAS) identified 60 loci associated with birth weight and concluded that 15% of the variance in birth weight can be explained by fetal genetic variation^13^. Similarly, a number of genetic association studies have identified single gene variants associated with abnormal fetal growth. Many of these variants are additionally known to be associated with type 2 diabetes (T2D), maturity-onset diabetes of the young (MODY) subtypes, or rare congenital diseases leading to metabolic alterations^13–16^. Most studies have used birth weight as a surrogate of intrauterine growth and have not assessed fetal genetic effects during specific gestational growth periods. Therefore, little is known on how fetal genetic variants may affect intrauterine growth, and if genetic variants have specific gestational periods, in which they are more important for fetal growth. Thus, we aimed to evaluate the prenatal effect of the recently GWAS identified birth weight variants by testing the impact of variants combined in a genetic risk score (GRS) on overall fetal growth during 2^nd^ and 3^rd^ trimester and independently during specific gestational weeks of fetal development. Subsequently, we hypothesized that the variants with the largest effect on fetal intrauterine growth, in this study, may represent essential growth pathways that could provide insight into the well-established link between intrauterine growth and adult disease. We therefore explored if the variants associated with fetal intrauterine weight were also associated with development of hypertension, T2D and BMI in adulthood using data from the UK Biobank.

## Results

Prenatal data from 665 newborn children were included in the analyses. The children were born of mothers with mean ± SD age of 30.23 ± 4.70 years and a pre-pregnancy BMI of 24.78 ± 4.93 kg/m^2^ (Table 1). The children had a mean birth weight of 3580 ± 474 g and were born at a mean gestational age of 279 ± 10 days (Table 1).

**Table 1:** Characteristics of pregnancies (n = 665).

*Maternal characteristics*	
Maternal age (years)	30.2 (4.7)
Maternal pre-pregnancy BMI (kg/m^2^)	24.8 (4.9)
Normal weight (BMI ≤ 25) n (%)	406 (61%)
Overweight (25 > BMI ≤ 30) n (%)	159 (24%)
Obese (BMI > 30) n (%)	85 (13%)
Mother smoking (yes/no)	62 (10%)
*Parity*:	
0	254 (38%)
1	282 (42%)
2	96 (14%)
3	17 (3%)
4	4 (0.6%)
*Ultrasound scans*:	
Fetal weight in grams at 20 weeks (mean ± SD)	328 ± 42.6
Fetal weight in grams at 25 weeks	893 ± 125
Fetal weight in grams at 32 weeks	2089 ± 250
*Delivery*:	
Gestational age in days	279 ± 10
Vaginal delivery	577 (87%)
Cesarean section	85 (13%)
Emergency	51
Elective	34
*Offspring characteristics*	
Gender (Male/Female)*	338/317
Birth weight (g)	3580 (474)
*Offspring with a low GRS (2.5*^*th*^ *percentile) (n* = *17)*	
Birth weight (g)	3310 (420)
Length (cm)	51.5 (1.6)
Maternal age	28.8 (4.1)
Maternal BMI (kg/m^2^)	25.5 (4.2)
*Offspring with a high GRS (97.5*^*th*^ *percentile) (n* = *17)*	
Birth weight (g)	3785 (356)
Length (cm)	52.5 (1.5)
Maternal age	31.4 (4.9)
Maternal BMI (kg/m^2^)	25.3 (4.8)

Data is expressed as mean (SD)/median (SD) or number of individuals in a group (%). Parity indicates the number of previous pregnancies carried to term. *Missing gender in 10 pregnancies. GRS: Genetic Risk Score.

**Figure 1:**
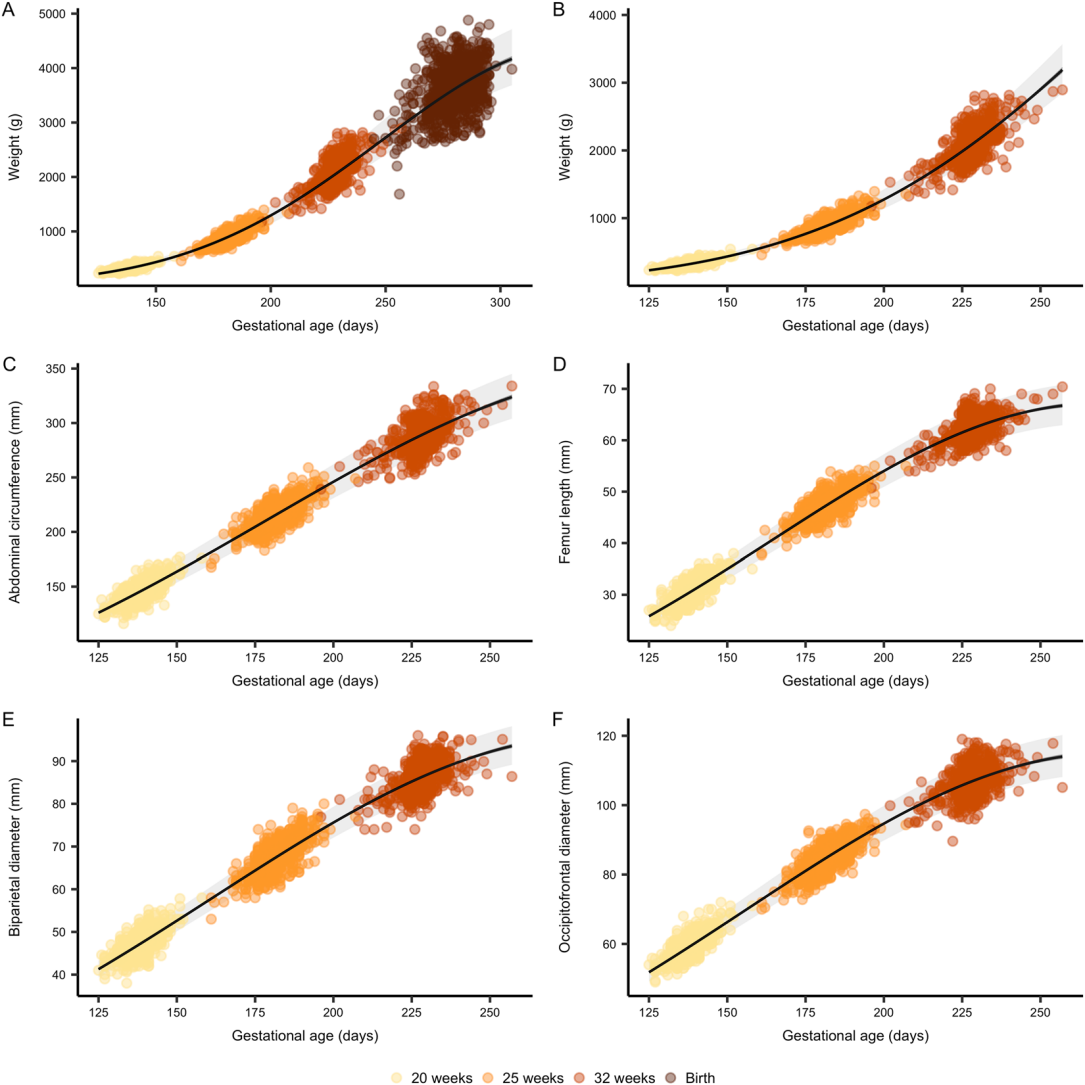
Fetal growth curves. Growth curves of fetal weight estimated by ultrasound with (**A**) and without (**B**) birth weight, as well as abdominal circumference (**C**), femur length (**D**), biparietal diameter (**E**) and occipito-frontal diameter (**F**) measured by ultrasound. Gestational age is estimated at the nuchal translucency scan (11–13 weeks of gestation) and used as reference throughout pregnancy. Lines represents the mean trend with 95% confidence (dark grey) and prediction intervals (light grey) from unconditional growth models fitted using mixed linear regression.

Fetal weight estimated by ultrasound at 20, 25, and 32 weeks of gestation displayed a curvilinear trajectory similar to previous reports of intrauterine growth^17–19^ (Fig. 1B), with a 4.7 (95%CI: 4.6, 4.7) % basal daily weight increment (linear time effect) and a 0.014 (95%CI: 0.013, 0.014) percentage point per day reduction in the daily proportional weight gain (quadratic time effect). The growth trajectory including weight measured at birth showed a similar trend with a 4.0 (3.9, 4.1) % basal daily weight increment and a 0.010 (95%CI: 0.010, 0.011) percentage point per day reduction in the daily proportional weight gain (Table 2).

**Table 2:** Unconditional fetal growth.

	Intercept		GA		GA^2^	
	β	g or mm	β	%·d^−1^	β	%-points·d^−1^
Weight (incl. birth weight)	0.774 (0.729; 0.819)	2.17 (2.07; 2.27)	0.0456 (0.0452; 0.0460)	4.67 (4.62; 4.71)	−6.82·10^−5^ (−6.93·10^−5^; −6.72·10^−5^)	−0.0136 (−0.0139; −0.0134)
Weight (excl. birth weight)	1.30 (1.22; 1.39)	3.68 (3.39; 4.01)	0.0395 (0.0385; 0.0404)	4.03 (3.93; 4.12)	−5.12·10^−5^ (−5.37·10^−5^; −4.86·10^−5^)	−0.0102 (−0.0107; −0.00972)
Occipitofrontal diameter	2.04 (2.00; 2.08)	7.69 (7.39; 7.99)	0.0198 (0.0194; 0.0202)	2.00 (2.00; 2.04)	−3.62·10^−5^ (−3.74·10^−5^; −3.50·10^−5^)	−0.00724 (−0.00748; −0.00670
Abdominal circumference	2.95 (2.90; 2.99)	19.1 (18.2; 20.0)	0.019 (0.0185; 0.0195)	1.92 (1.86; 1.97)	−3.1·10^−5^ (−3.24·10^−5^; −2.96·10^−5^)	−0.0062 (−0.00648; −0.00591)
Femur length	0.861 (0.819; 0.904)	2.37 (2.27; 2.47)	0.0249 (0.0244; 0.0254)	2.52 (2.47; 2.57)	−4.64·10^−5^ (−4.77·10^−5^; −4.51·10^−5^)	−0.00928 (−0.00954; −0.00903)
Biparietal diameter	1.89 (1.85; 1.93)	6.62 (6.39; 6.87)	0.0187 (0.0183; 0.0191)	1.89 (1.85; 1.93)	−3.28·10^−5^ (−3.39·10^−5^; −3.17·10^−5^)	−0.00657 (−0.00679; −0.00635)

Fetal growth was modeled using linear mixed regression. Data is presented as raw parameter estimates (**β**) and estimates transformed to the original scale with respective 95% confidence intervals. Intercept represents the mean (geometric) at zero days of gestation. GA (gestational age) represent the daily proportional gain at zero days of gestation. GA^2^ represent the change in daily proportional gain per day.

Abdominal circumference, biparietal diameter, occipitofrontal diameter and femur length measured by ultrasound also displayed curvilinear growth with comparable trajectories and daily proportional increments ranging from 1.9 to 2.5% per day and a reduction in the daily proportional increment ranging from 0.006 to 0.009 percentage points per day (Table 2, Fig. 1).

### Genetic risk score of birth weight associates with intrauterine weight and fetal girth

**Figure 2:**
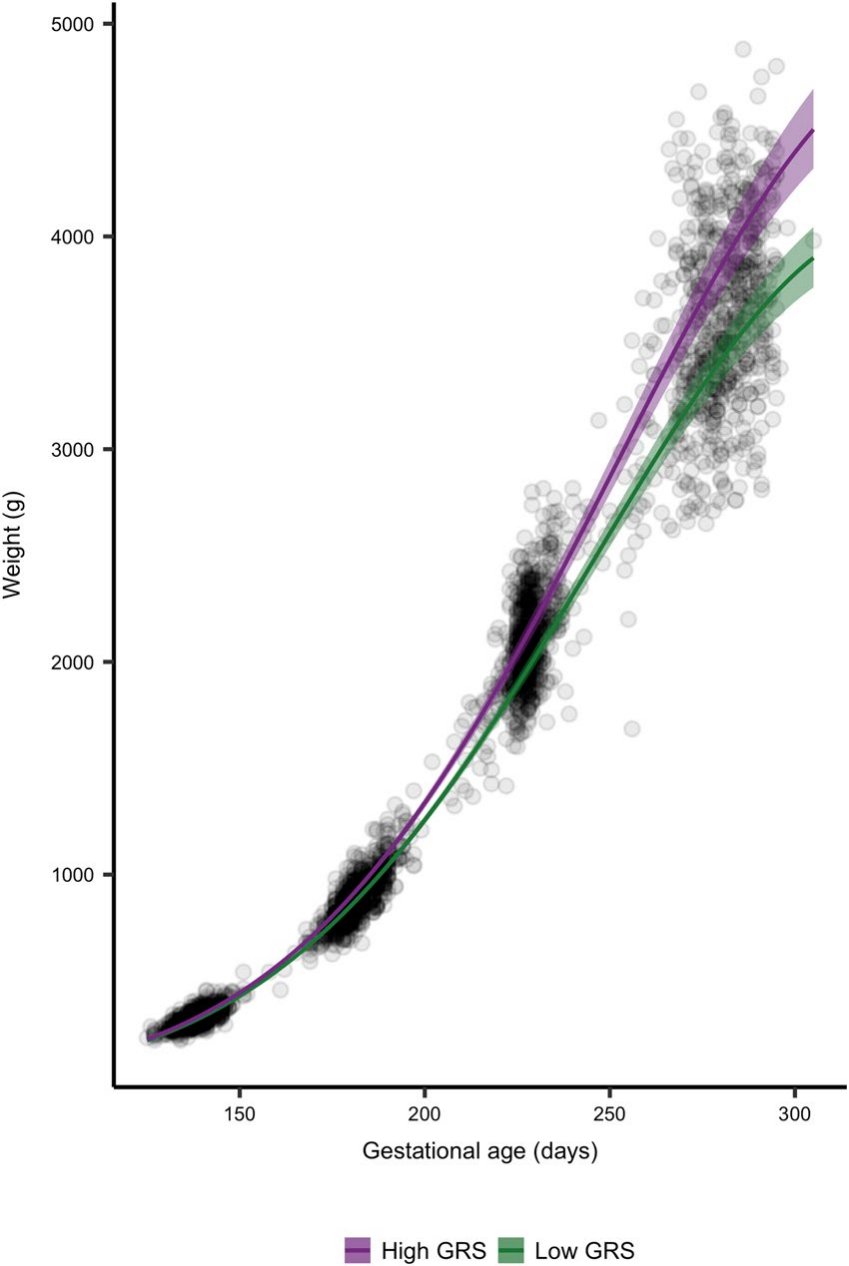
Fetal growth according to high and low GRS. Growth curves of fetal weight estimated by ultrasound and birth weight. Gestational age was estimated at the nuchal translucency scan (11–13 weeks of gestation) and used as reference throughout pregnancy. Lines represent the mean trend with 95% confidence intervals from conditional growth models fitted using mixed linear regression.

We tested the effect of an unweighted birth weight-raising GRS on intrauterine growth using mixed linear regression models. We found a significant effect of the GRS, which attenuated the reduction in the daily proportional weight gain by 7.78 × 10^−6^ (95%CI: 1.64 × 10^−6^, 1.33 × 10^−5^) percentage points per day per birth weight-raising allele (Table 3). The association was stronger when including weight measured at birth showing a per allele effect of 8.92 × 10^−6^ (95%CI: 4.32 × 10^−6^, 1.4 × 10^−5^) percentage points per day, corresponding to a mean difference at term of 410 g (95%CI: 115, 798) g between a child with the lowest and highest GRS (Fig. 2).

**Table 3:** Fetal growth conditioned on genetic risk.

	Adjusted	GA^2^ × GRS			GA^2^ × BMI			GA^2^ × Sex		
		β	%-points·d^−1^·allele^1^	P	β	%-points·d^−1^·m^2^·kg^−1^	P	β	%-points·d^−1^	P
Weight (incl. birth weight)	No	5.71·10^−5^ (7.21·10^−6^; 1.07·10^−4^)	1.42·10^−2^ (1.44·10^−3^; 2.1·10^−2^)	0.025						
	Yes	4.46·10^−8^ (2.16·10^−8^; 6.98·10^−8^)	8.92·10^−6^ (4.32·10^−6^; 1.40·10^−5^)	2.0·10^−4^	4.1·10^−8^ (1.87·10^−8^; 6.24·10^−8^)	8.2·10^−6^ (3.74·10^−6^; 1.25·10^−5^)	3.3·10^−4^	−4.99·10^−7^ (−7.13·10^−7^; −2.85·10^−7^)	−9.98·10^−5^ (−1.43·10^−4^; −5.7·10^−5^)	4.7·10^−6^
Weight (excl. birth weight)	No	3.35·10^−5^ (1.34·10^−6^; 6.57·10^−5^)	6.7·10^−3^ (2.68·10^−4^; 1.3·10^−2^)	0.041						
	Yes	3.74·10^−8^ (8.21·10^−9^; 6.65·10^−8^)	7.78·10^−6^ (1.64·10^−6^; 1.33·10^−5^)	0.012	4.21·10^−8^ (1.58·10^−8^; 6.65·10^−8^)	8.42·10^−6^ (3.16·10^−6^; 1.33·10^−5^)	0.0017	−4.53·10^−7^ (−7.1·10^−7^; −1.95·10^−7^)	−9.06·10^−5^ (−1.4·10^−4^; −3.9·10^−5^)	5.9·10^−4^
Occipitofrontal diameter	No	4.13·10^−7^ (−7.14·10^−7^; 1.54·10^−6^)	8.3·10^−5^ (−1.43·10^−4^; 3.08·10^−4^)	0.470						
	Yes	4.29·10^−9^ (−7.09·10^−9^; 1.57·10^−8^)	8.58·10^−7^ (−1.42·10^−6^; 3.14·10^−6^)	0.460	1.20·10^−8^ (1.69·10^−9^; 2.22·10^−8^)	2.4·10^−6^ (3.38·10^−7^; 4.44·10^−6^)	0.022	−3.27·10^−7^ (−4.27·10^−7^; −2.26·10^−7^)	−6.54·10^−5^ (−8.54·10^−5^; −4.52·10^−5^)	2.2·10^−10^
Abdominal circumference	No	4.49·10^−6^ (1.11·10^−6^; 7.88·10^−6^)	8.9·10^−4^ (2.22·10^−4^; 15.76·10^−4^)	0.009						
	Yes	1.61·10^−8^ (3.01·10^−9^; 2.91·10^−8^)	3.22·10^−6^ (6.02·10^−7^; 5.82·10^−6^)	0.016	1.3·10^−8^ (1.41·10^−9^; 2.50·10^−8^)	2.6·10^−6^ (2.82·10^−7^; 5·10^−6^)	0.028	−1.69·10^−7^ (−2.84·10^−7^; −5.29·10^−8^)	−5.07·10^−5^ (−5.68·10^−5^; −1.06·10^−5^)	0.0043
Femur length	No	4.23·10^−7^ (−1.92·10^−7^; 1.04·10^−6^)	8.46·10^−5^ (−3.84·10^−6^; 2.08·10^−6^)	0.178						
	Yes	4.23·10^−7^ (−1.92·10^−7^; 1.04·10^−6^)	8.46·10^−5^ (−3.84·10^−5^; 2.08·10^−4^)	0.178	8.8·10^−7^ (3.31·10^−7^; 1.44·10^−6^)	1.7·10^−4^ (6.6·10^−5^; 2.9·10^−6^)	0.0018	1.97·10^−8^ (−3.48·10^−6^; 7.42·10^−6^)	3.94·10^−6^ (−6.96·10^−4^; 1.5·10^−3^)	0.480
Biparietal diameter	No	5.33·10^−7^ (−4.55·10^−7^; 1.52·10^−6^)	1.66·10^−4^ (−9.1·10^−5^; 3.04·10^−4^)	0.290						
	Yes	5.95·10^−9^ (−6.02·10^−9^; 1.79·10^−8^)	1.19·10^−6^ (−1.20·10^−6^; 3.58·10^−6^)	0.330	9.64·10^−9^ (−1.15·10^−9^; 2.04·10^−8^)	1.93·10^−6^ (−2.3·10^−7^; 4.08·10^−6^)	0.080	−3.14·10^−7^ (−4.2·10^−7^; −2.08·10^−7^)	−6.28·10^−5^ (−8.4·10^−5^; −4.15·10^−5^)	6.8·10^−9^

Data is presented as raw parameter estimates (**β**) and estimates transformed to the original scale with respective 95% confidence intervals and corresponding P values. Fetal growth conditioned on genetic birth weight risk score (GRS) with or without co-conditioning on maternal pre-pregnancy body mass index (BMI) and fetal sex was modelled using linear mixed regression. GA^2^ × GRS represents the additive per allele effect of the GRS on change in daily proportional weight gain per day. GA^2^ × BMI represent the additive effect of a one unit (kg·m^2^) increment in BMI on change in daily proportional weight gain per day. GA^2^ × Sex represent the additive effect of female fetal sex on change in daily proportional weight gain per day.

Fetal weight estimated by ultrasound is a compound measure based on femoral length, abdominal circumference and head circumference, the latter itself based on biparietal diameter and occipitofrontal diameter. When testing the impact of the GRS on each of these individual measurements we found a 3.22 × 10^−6^ (95%CI: 6.02 × 10^−6^, 5.82 × 10^−5^) percentage point lower reduction in daily proportional increase in abdominal circumference per birth weight-raising allele (Table 3).

We tested the impact of the GRS on specific fetal growth periods, week 20–25, week 25–32 and week 32-birth by multiple linear regression models adjusted for gestational age, weight and gestational age at previous ultrasound scan, maternal pre-pregnancy BMI and fetal gender. We found an association between the GRS and fetal growth in the period week 20–25 (β = 0.94 ± 0.60 g per day per birth weight-raising allele, p = 0.011), and in the period week 25–32 (β = 1.75 ± 1.45 g per day per birth weight-raising allele, p = 0.023), an additionally a strong association in the period week 32 to birth (β = 8.10 ± 2.99 g per day per birth weight-raising allele, p = 0.007).

We tested if the GRS was associated with abnormal fetal weight measured as either LGA or SGA. We found no association between the GRS and LGA (OR 1.07 (95%CI: 0.98, 1.17)) and similarly, no association between the GRS and SGA was found (OR 0.96 (95%CI: 0.90, 1.02)).

### Associations and interactions between maternal traits and GRS on intrauterine growth and birth weight

We hypothesized that maternal environmental traits such as smoking, BMI and glucose levels might influence the effect of the GRS, and tested if there were any interactions between the additive per allele effect of the GRS on change in daily proportional weight gain per day (GA^2^ × GRS) and the given traits. There was no interaction between maternal pre-pregnancy BMI and intrauterine growth (p = 0.47) or birth weight (p = 0.45), and similarly no interactions when testing across tertiles of the GRS (p = 0.89).

However, we did observe marginal effects of maternal pre-pregnancy BMI on several fetal growth parameters (Table 3). Maternal BMI weakened the reduction in the daily proportional weight gain by 8.2 × 10^−6^ (95%CI: 3.74 × 10^−6^, 1.25 × 10^−5^) percentage points per day per birth weight-raising allele (Table 3). Same effects were seen for fetal AC, OFD and femur length growth, but no effect on BPD.

We assessed the effect of co-conditioning of maternal BMI on fetal growth (including birth weight) and observed a small effect of BMI on the attenuation of the daily proportional weight gain by 5.01 × 10^−5^ (95%CI: −1.1 × 10^−5^, 1.1 × 10^−4^) percentage points per day per birth weight-raising allele compared to 8.92 × 10^−6^ (95%CI: 4.32 × 10^−6^, 1.40 × 10^−5^) without co-conditioning of BMI.

We then tested if maternal glucose levels were interacting with the GRS. We had measures of 2 hour glucose levels from oral glucose tolerance tests (OGTTs) in 155 women and included the interaction term (GA^2^ × GRS × glucose) in the mixed linear regression model of intrauterine growth. We found no interaction of maternal glucose levels on intrauterine weight (p = 0.76) or birth weight (p = 0.90).

Sixty-two of the mothers were smoking during pregnancy. We tested the interaction (GA^2^ × GRS × smoking) and found no effect of maternal smoking on intrauterine weight (p = 0.82) or birth weight (p = 0.10).

### Genetic variants associated with intrauterine weight

We tested the independent effect of all variants included in the GRS on intrauterine weight (Suplementary Table 1). Eight variants were associated with overall intrauterine growth. We then tested the impact of variants in growth periods from 20–25 weeks, from 25–32 weeks and from 32 weeks to birth. Two variants (rs61830764 and rs61862780) showed association with fetal growth in the period week 20–25. Similarly, two variants (rs6989280 and rs28510415) were associated with fetal growth in week 25–32, while four variants were associated with fetal growth from week 32 to birth (Suplementary Table 1).

### Genetic variants associated with adult onset disease

The eight variants, which were associated with intrauterine fetal growth, were tested for association with T2D, hypertension and BMI in the UK Biobank (Table 4). This was done to test, if the variants with the largest genetic effect on intrauterine growth trajectories would have an effect on development of adult metabolic disease. The variant, rs700059, which was positively associated with overall intrauterine growth, was inversely correlated with T2D in a subsample of 19,630 individuals in the UK Biobank (β = −0.002 ± 0.001, p = 0.0007). Same direction of effect was reported in the DIAGRAM GWAS of 152,000 individuals (p = 0.032), but not reaching the GWAS significance level^20^. In contrast, rs10830963, showed a strong positive association with development of T2D (β = 0.003 ± 0.001, p = 6.37 × 10^−11^), with the same direction of effect in the DIAGRAM GWAS (p = 1.7 × 10^−7^). We tested for association with BMI and found an inverse association with rs700059 (β = −0.030 ± 0.014, p = 0.026), and a positive associations with rs10830963 (β = 0.028 ± 0.010, p = 0.008) and rs72851023 (β = 0.051 ± 0.018, p = 0.004). The GIANT GWAS of 339,224 individuals has previously reported same direction of effect for association between variants and BMI (p = 0.0005, p = 0.069 and p = 0.012, respectively), but none of them reaching the GWAS significance threshold (p < 5 × 10^−8^)^21^.

**Table 4:** Association of genetic variants with adult onset disease in the UK biobank.

Variant	EA	Type 2 diabetes (n = 19.630)	P value	Hypertension (n = 77,533)	P value	BMI (n = 490,000)	P value
		β ± SE		β ± SE		β ± SE	
rs7964361	C	−0.000 ± 0.001	0.935	0.001 ± 0.001	0.554	0.030 ± 0.017	0.067
rs61154119	T	−0.001 ± 0.001	0.129	−0.000 ± 0.001	0.827	0.009 ± 0.013	0.473
rs1374204	T	0.000 ± 0.001	0.412	−0.001 ± 0.001	0.198	0.020 ± 0.01	0.060
rs72851023	T	−0.001 ± 0.001	0.060	−0.003 ± 0.002	0.091	0.051 ± 0.018	**0**.**004**
rs700059	G	−0.002 ± 0.001	**0**.**0007**	−0.002 ± 0.001	**0**.**034**	−0.030 ± 0.014	**0**.**026**
rs10830963	G	0.003 ± 0.001	**6**.**37E-11**	−0.000 ± 0.001	0.643	0.028 ± 0.010	**0**.**008**
rs138715366	C	0.002 ± 0.002	0.490	−0-004 ± 0.004	0.366	0.025 ± 0.050	0.613
rs2150052	T	−0.001 ± 0.000	0.206	−0.002 ± 0.001	**0**.**039**	−0.005 ± 0.009	0.600

Analyses assessing the effect of genetic variants associated with intrauterine growth in this study and their impact on adult onset type 2 diabetes, hypertension and BMI in the UK biobank of 19,630–490,000 individuals. Occurrence of Type 2 diabetes and hypertension are based on ICD-10 codes. BMI is measured at inclusion in the UK biobank. EA: Effect allele. P values are calculated by linear regression or logistic regression models.

## Discussion

In this study, we evaluated the combined effect of GWAS-identified birth weight-raising variants on intrauterine fetal growth from 20 gestational weeks to birth. We demonstrate that the birth weight-raising GRS was associated with overall intrauterine fetal growth, suggesting that the fetal genetic contribution to birth weight is mediated throughout pregnancy. We found a difference in birth weight of 410 g in children with low and high GRS, indicating that the combined effect of genetic variants has a marked effect on intrauterine growth, which is measurable at birth. We found eight independent variants with positive effects on intrauterine fetal growth, and our findings suggest that some variants are associated with overall intrauterine weight, while others seem to act in specific gestational time windows. We used an explorative approach to test if the variants, associated with intrauterine growth in this study, were associated with adult metabolic disease in the UK Biobank. We demonstrate that, in particular, two of the variants, rs700059 and rs10830963, are associated with T2D, hypertension or BMI in the UK Biobank. These findings emphasize the importance of the fetal genetic contribution to intrauterine growth, which may provide the basis for further understanding of the link between intrauterine growth and risk of later metabolic disease.

Birth weight has previously been used for assessment of fetal intrauterine growth, since it is a simple measure, obtained on most individuals at birth, and easy to compare across cohorts. However, birth weight does not give comprehensive information on growth trajectories during pregnancy. In this study, we used ultrasonic measures of fetal weight, which enabled us to draw a detailed picture of the impact of genetic variants on fetal growth at specific time points during pregnancy. We found a significant effect of the GRS on overall intrauterine growth from 20 weeks of gestation to birth (p = 2.0 × 10^−4^) corresponding to an attenuation of the reduction in daily proportional weight gain by 8.92 × 10^−6^ percentage points per day per birth weight-raising allele. We hypothesized that maternal environmental factors might influence the effect of the GRS and found a marginal independent effect of maternal pre-pregnancy BMI. Previous studies, have assessed the impact of maternal BMI on fetal growth, and it is well-established that maternal obesity increases the risk of a large-for-gestational age fetus^19,22^. Similarly, greater fetal biometries can be detected in obese women as early as 21 weeks of gestation and estimated fetal weight is progressively greater from 30 weeks of gestation in compared to normal weight women^19^. However, despite these previous associations between maternal obesity and fetal growth, our study implies that maternal BMI only has a marginal effect on genetic growth trajectories of the fetus. The effect of maternal BMI on genetic growth trajectories could be mediated through a stronger genetic predisposition to obesity, which in the mother leads to a higher BMI, and cause a similar phenotype in the fetus with increased intrauterine growth and higher birth weight^23^.

When assessing the impact of fetal genetics on intrauterine growth, it is important to acknowledge that 50% of genes are shared between mother and fetus. In general, the maternal genotypes contribute relatively little to birth weight^13,24,25^, unless the maternal genotype directly influences the intrauterine environment^14,15^. This has been demonstrated in carriers of rare heterozygous glucokinase *(GCK)* mutations, which causes altered pancreatic glucose-sensing. A fetal *GCK* mutation causes a ~530 g decrease in birth weight, whereas a maternal *GCK* mutation causes a ~600 g increase in birth weight; but if the *GCK* mutation is present in both mother and fetus, no effect on birth weight is observed^15^. A similar effect has been observed for *TCF7L2*, a mutation in which causes an increase in birth weight when carried by the fetus and/or mother^14^; an effect likely mediated by elevated fasting plasma glucose levels. Our results do not indicate that the combined genetic effect of birth weight variants on intrauterine growth is mediated or modified by stimulated plasma glucose. However, it is possible that maternal obesity-related traits could lead to higher birth weight regardless of fetal genotype as we see in the independent effect of BMI on intrauterine growth^26,27^.

We identified eight variants positively associated with intrauterine growth during pregnancy. All eight variants were exerting their effect on fetal growth throughout pregnancy and not in specific gestational weeks. In contrast, two variants (rs61830764 and rs61862780) showed association with fetal growth in the period week 20–25, suggesting that these may be more important for fetal growth in the second trimester of pregnancy. Similarly, two variants (rs6989280 and rs28510415) were associated with fetal growth in week 25–32, indicating that their effect is most important in late second trimester and early third trimester.

The variant, rs7964361 had the largest effect size, attenuating the unconditional reduction in daily proportional weight gain and is located in the insulin-like growth factor 1 (*IGF1*) gene, which encodes the insulin-like growth factor 1 hormone (IGF1). In mice, maternal IGF1 has been shown to stimulate fetal growth by increasing the transfer of nutrients across the placenta *in vivo*^28,29^, while *in vitro* studies have suggested that fetal IGF1 stimulates fetal growth^30^. Concurrently, ablation or differential methylation of *IGF1* has been shown to cause abnormal fetal growth in mice^29,31^. In line with these findings, our results suggest that rs7964361 may play a role in fetal intrauterine growth.

A number of observational studies have highlighted the importance of the intrauterine environment in relation to disease risk later in life, such as development of T2D. The association between birth weight and T2D is mediated through both non-genetic and genetic factors, among which the genetic effects during fetal life may play a substantial role^13,32^. A number of common genetic variants have been associated with both birth weight and T2D^13^; however, only a small proportion of the heritability of both traits can be explained by these variants^13,32^. We hypothesized that the variants with the largest effect on fetal intrauterine growth, in this study, may represent essential growth pathways that could provide insight into the well-established link between intrauterine growth and adult disease^7,8^. We found an association between intrauterine growth and rs10830963; a variant which is strongly associated with T2D in the UK Biobank (p = 6.37 × 10^−11^). rs10830963 is an intronic variant located in the Melatonin receptor 1B gene (*MTNR1B*), which encodes one of the two receptors of melatonin, a hormone involved in circadian rhythms. Previous studies have reported a strong effect of rs10830963 on fasting glucose levels, insulin secretion and disposition index^33–35^, as well as risk of T2D identified through large GWAS^36,37^. Similarly, GWAS significant association of rs10830963 and offspring birth weight in maternal carriers have recently been reported^38^. The association between maternal rs10830963 and birth weight is likely mediated through elevated maternal fasting glucose levels, causing increased placental transfer of glucose, which leads to higher fetal insulin secretion and thereby increased birth weight. Our study suggests that the effect of the fetal variant also leads to increased fetal intrauterine growth. However, whether the effect is caused by maternal transmission of the variant, is mediated through altered maternal glucose levels^38^ or if the variant affects fetal glucose regulation per se is unknown. Further studies are needed to investigate this.

There are some limitations to this study. First of all, we used an unweighted GRS comprised of GWAS-identified birth weight variants^13^, that assumes an additive and equal effect of all variants at each time point during pregnancy, which may not be completely accurate. We chose this method, because we expect the effect sizes to vary substantially from second to third trimester and the reported effect estimates are based on birth weight alone^13^. However, using this method does not take into account any interactions between variants or genes that may be present. Additionally, we have used a cohort of 665 children, which for genetic studies may be of relatively small size and of limited power to detect associations. However, we used birth weight variants identified by GWAS, so it is thus plausible that these variants should be associated during prenatal growth without a strict statistical threshold. Another limitation to this study is that we used fetal weight calculated by the Hadlock formula as an estimation of fetal growth, and there may be some inaccuracies in the estimations. In particular, we expect to have some uncertainties in weight estimations in the third trimester, where it is difficult to achieve precise measures of the fetal head and abdominal circumferences. However, we used the same sonographers throughout the study period, which may limit the intra-observer and inter-observer variations. We did not have data on maternal genotypes in this study, which would have made it possible to assess how maternal genotypes would affect fetal growth and adjust our analyses accordingly. In line with this, detailed information and adjustment for maternal environmental factors such as diet and gestational weight gain would have strengthened the analyses.

In conclusion, we demonstrate that a GRS of birth weight-raising variants is associated with intrauterine fetal growth suggesting that the fetal genetic contribution to birth weight is mediated throughout pregnancy. We show that some variants influence fetal growth in specific gestational weeks and may be more important for fetal growth in these time windows. Additionally, our study supports that variants associated with fetal intrauterine growth may also have an impact on development of metabolic disease later in life.

## Methods

Pregnant women (>18 years of age) attending a nuchal translucency scan at 12 weeks of gestation at Roskilde University Hospital were invited for participation in this study, as previously described^39,40^.

In addition to the nuchal translucency scan at 12 weeks of gestation, the women were invited for a malformation ultrasound scan at 20 weeks of gestation, and for fetal growths scans at 25 and 32 weeks of gestation. A total of 753 pregnancies were included in the study. Exclusion criteria were missing genotypes, maternal gestational diabetes, pre-eclampsia, and twins gestations, incomplete follow-up, inclusion of pregnancy twice, or presence of an acardiac twin, leaving 665 pregnancies in the study.

### Ultrasound scans

Ultrasound scans were performed on ultrasound scanners from GE Healthcare (Buckinghamshire, UK) by trained midwifes or medical doctors. Estimation of gestational age was based on ultrasound measurements of the fetal crown–rump length (CRL) at the nuchal translucency scan. Assessment of fetal weight were based on two-dimensional ultrasound and included measurements of abdominal circumference (AC) in mm, bi-parietal diameter (BPD) in mm, occipital-frontal diameter (OFD) in mm and length of the femur bone (FL) in mm. BPD was measured using an ultrasound probe in an axial plane, traversing the thalami, and cavum septum pellucidum with the transducer perpendicular to the central axis of the head. The distance from the outer edge of the near calvarial parietal wall to the inner edge of the far parital calvarial wall was termed the BPD, while distance from the near occipital wall to the far frontal calvarial wall was measured as the OFD. FL was measured as the longest length of the femoral bone in a horizontal plane. AC was measured as the transverse abdominal circumference at the level of the fetal ventricle and portal vein.

Fetal weight in grams was calculated based on the Hadlock formula^18^:

$$\mathrm{Weight}(g)={10}^{(1.326-0.00326\times {\rm{AC}}\times {\rm{FL}}/100)+(0.0107\times {\rm{HC}}/10)+(0.0438\times {\rm{AC}}/10)+(0.158\times {\rm{FL}}/10)).}$$

The definition of small-for-gestational-age (SGA) was a newborn child with a birth weight below −15% (<10^th^ percentile) for gestational age at birth. The definition of large-for-gestational-age (LGA) was a child with a birth weight >22% (>90^th^ percentile) for given gestational age at birth^41,42^.

### Pregnancy outcome

The outcome of the pregnancy was retrieved from patients’ medical records including data on birth weight, mode of delivery and gender of the baby. Parity was defined as birth of a baby after 22 weeks of gestation and registered as 0–5. Women who had not previously given birth (parity = 0), women who had given birth once (parity = 1), twice (parity = 2), three times (parity = 3) or four times (parity = 4) prior to the examined pregnancy.

### Anthropometric measurements

All women reported their age, pre-pregnancy weight and height at the first visit. BMI was calculated as BMI = weight (kg)/height^2^ (m). Additionally, the mothers reported if they were smoking or not during pregnancy. If they had any medical diseases before or during pregnancy it was reported. Similarly if the women were treated with medication during pregnancy it was recorded.

### Biochemical measures

Women with a pre-pregnancy body mass index (BMI) ≥ 27 kg/m^2^, family history of diabetes, glucosuria or previous birth of baby weighing ≥ 4500 g were offered an oral glucose tolerance test (OGTT) according to clinical practice in Denmark. The OGTT was performed at 14–20 weeks if the women had two risk factors or previous GDM or at 28–30 weeks of gestation if the woman had only one risk factor. The OGTT was based on intake of 75 g glucose after a minimum 10-hour overnight fast, with plasma glucose concentration measured two hours after ingestion.

Acid-base testing of the cord blood was performed following delivery in both vaginal and cesarean deliveries. After the sample for acid-base testing was obtained, a sample of cord blood was taken for genotyping. Due to this procedure, it was not possible to obtain blood for genotyping in all children due to either clotting of the cord vessels, not enough blood for sample, or unawareness from the midwife/doctor. In total 753 cord blood samples were retrieved and kept in a −80 °C freezer until DNA extraction.

### Genotyping and QC

We used DNA from cord blood samples from 753 children for genotyping using the Illumina Infinium HumanCoreExome Beadchip platform (Illumina, San Diego, CA). Genotypes were called using the Genotyping module (version 1.9.4) of GenomeStudio software (version 2011.1, Illumina). We excluded, duplicates, ethnic outliers, and samples with extreme inbreeding coefficients, mislabeled or missing sex, or call rate <95%, leaving 701 children who passed all quality control criteria.

### Genetic risk score

The genetic variants selected for the GRS were based on 60 GWAS-identified birth weight-raising loci^13^. Variants or proxies (*r*^2^ ≥ 0.90) for the 60 loci associated with BW were retrieved from the Human ExomeBeadChip. Two loci were not present on the array; rs139975827 was not captured by any proxy and rs11096402 was located on the X-chromosome, which was not imputed. Hence, we included 58 loci in an unweighted GRS, which was constructed by summing the dosages for each birth weight-raising allele. The GRS ranged from 47.5 to 78.8 alleles and was normally distributed. The cut-offs for tertiles were based on the distribution of GRS. The cut-offs for tertiles were GRS < 53.1 (1^st^ tertile), GRS 53.1–56.6 (2^nd^ tertile), GRS > 56.6 (3^rd^ tertile).

### Statistical analyses

Data analysis was performed in R version 3.4.1 (www.r-project.org). Two-tailed testing was used and statistical significance was defined as a two-sided p-value below 0.05.

### Overall intrauterine growth

To model the curvilinear increase in fetal weight estimates from week 20 to birth, we fitted an unconditional growth model with a linear and quadratic time trend, using mixed linear regression with a random intercept and random growth trajectory of each fetus:

1 $${\rm{weight}} \sim {\rm{GA}}+{{\rm{GA}}}^{2}$$

To assess the impact of the GRS on overall intrauterine growth, an interaction with the quadratic time term was added, thereby conditioning the change in weight over time on the GRS:

2 $${\rm{weight}} \sim {\rm{GA}}+{{\rm{GA}}}^{2}+{{\rm{GA}}}^{2}\times {\rm{GRS}}$$

A fixed effect interactions with the linear time component was not specified, as this represents the initial growth rate at time = 0; too early for the GRS to have any measurable biological effect. In order to adjust for effects of fetal sex and maternal pre-pregnancy BMI, interactions between these covariates and the quadratic time term were added to the conditional model:

3 $${\rm{weight}} \sim {\rm{GA}}+{{\rm{GA}}}^{{\rm{2}}}+{{\rm{GA}}}^{{\rm{2}}}\times {\rm{GRS}}+{{\rm{GA}}}^{{\rm{2}}}\times {\rm{Sex}}+{{\rm{GA}}}^{{\rm{2}}}\times {\rm{BMI}}$$

Fetal weight in gram was log-transformed prior to analysis to address the issue of increasing heteroscedasticity of weight measurements during pregnancy. Models were fitted by restricted maximum likelihood using a Gaussian spatial structure to model the correlation between temporally non-equidistant weight estimates. Model assumptions were assessed visually by inspection of normal probability plots and residual plots.

To assess the impact of the individual genetic variants on overall intrauterine growth, similar conditional growth models with and without adjustment for fetal sex and maternal pre-pregnancy BMI were fitted. Models of GRS and individual variant effects were fitted with and without birth weight measurements to assess whether birth weight was the sole driver of identified associations.

Interactions between the GRS and maternal environment traits such as BMI, smoking and glucose levels were tested by including the respective interaction terms in the model (i.e. GA^2^ × GRS × BMI).

### Specific growth periods

To assess the impact of the GRS during specific gestational growth periods, we used multiple linear regression models. For instance, to assess the impact on growth from the malformation scan at week 20 to the first study-specific scan at 25 weeks of gestation, we specified a model describing weight at 25 weeks as a function of the GRS, including weight at 20 weeks to adjust for growth prior to the period in question and actual gestational age at both measurements to adjust for variation in the actual age at which the ultrasound scans were performed (Weight_25weeks_ ~ GRS + Weight_20weeks_ + GA_20weeks_ + GA_25weeks_).

Growth from 25 to 32 weeks and from 32 weeks to birth was modeled in the same way. In order to adjust for effects of fetal sex and maternal pre-pregnancy BMI, main effects of these covariates were added to the model (Weight_25weeks_ ~ GRS + Weight_20weeks_ + GA_20weeks_ + GA_25weeks_ + Sex + BMI).

To assess the impact of individual variants during specific gestational growth periods, similar models with and without adjustment for fetal sex and maternal pre-pregnancy BMI were fitted. No correction for multiple testing was performed, since these loci were previously identified for association with BW, and it is therefore plausible to assume that they are associated with fetal growth.

### Birth weight

The impact of maternal pre-pregnancy BMI on birth weight was assessed by simple linear regression, whereas multiple linear regression adjusted for fetal sex, maternal pre-pregnancy BMI and gestational age at birth was applied to test for associations between the GRS and birth weight.

### Adult disease in UK Biobank

We used birth weight-raising variants in the GRS to test for association with intrauterine growth. The variants have previously been tested for association with T2D, hypertension and BMI in a birth weight GWAS^13^. In line with this, we wanted to test if the variants associated with intrauterine growth (and not only birth weight) were involved in development of metabolic disease using data from the UK Biobank of 490,000 individuals. We hypothesized that this would provide insight into the genetic aspects of fetal growth trajectories, and which direction of effect they have on later metabolic disease. The association between intrauterine growth variants and T2D or hypertension (ICD-10) was tested using logistic regression, while association with BMI measured at baseline was tested using simple linear regression. We additionally tested all direction of effects of associations with BMI in the GIANT GWAS summary statistics and for T2D in the DIAGRAM GWAS summary statistics^20,21^.

### Ethics

The study was approved by the Ethics Committee for Region Zealand (SJ-55 and SJ-347) and the Danish Data Protection Agency and was registered in ClinicalTrials.gov (Identifier: NCT00836524). The study was conducted in accordance with the principles of the Helsinki Declaration. All study participants provided written informed consent.

### Data Availability

The authors confirm that, for approved reasons, some access restrictions apply to the data underlying the findings. Data are available from the Novo Nordisk Foundation Center for Basic Metabolic Research, Section of Metabolic Genetics whose authors may be contacted.

## Electronic supplementary material

Supplementary information
