# Supplementary material for: Birth weight variants are associated with variable fetal intrauterine growth from 20 weeks of gestation
Source: Sci Rep. 2018 May 30;8:8376. doi: 10.1038/s41598-018-26752-3 (PMC5976727; doi:10.1038/s41598-018-26752-3)
Supplement: Supplementary file 1 — Supplementary information [file 41598_2018_26752_MOESM1_ESM.docx]

**Birth weight variants are associated with variable fetal intrauterine growth from 20 weeks of gestation.**

L. Engelbrechtsen^1,2^, D. Gybel-Brask^3^, Y. Mahendran^1,2^, M. Crusell^1^, T.H. Hansen^1^, TM Schnurr^1,2^, E. Hogdall^4^, L. Skibsted^3^, T. Hansen^1^, H. Vestergaard^1,5^

**Supplementary Table 1: Association between genetic variants and intrauterine weight**

| **Variants** | ***Nearest gene*** | **EA** | **Fetal growth week 20-25**  **(weight week 25)** | |  | **Fetal growth week 25-32**  **(weight week 32)** | |  | **Fetal growth week 32-birth**  **(weight at birth)** | |  | **Overall intrauterine weight** |  |
| --- | --- | --- | --- | --- | --- | --- | --- | --- | --- | --- | --- | --- | --- |
|  |  |  | **β g/day  (95% CI)** | **p value** | | **β g/day  (95% CI)** | **p value** | | **β g/day (95% CI)** | **p value** | | **β**  **%-points/day/allele** | **p value** |
| rs7964361 | *IGF1* | A | 0.48 (-10.18; 11.15) | 0.93 | | 20.61 (-4.86; 46.08) | 0.11 | | 35.47 (-17.10; 88.05) | 0.19 | | 8.84E-05 (2.80E-05; 1.49E-04) | **<0.01** |
| rs61154119 | *ACTL9* | T | 5.65 (-3.34; 14.63) | 0.22 | | 6.47 (-15.18; 28.11) | 0.56 | | -6.30 (-51.37; 38.76) | 0.78 | | 6.18E-05 (1.87E-05; 1.05E-04) | **0.01** |
| rs1374204 | *EPAS1* | T | 1.25 (-6.43; 8.93) | 0.75 | | -7.46 (-26.08; 11.16) | 0.43 | | 10.89 (-28.14; 49.93) | 0.58 | | 3.92E-05 (4.42E-06; 7.40E-05) | **0.03** |
| rs72851023 | *INS-IGF2* | T | 3.30 (-6.83; 13.44) | 0.52 | | -9.09 (-33.68; 15.51) | 0.47 | | 0.48 (-50.63; 51.59) | 0.99 | | 5.80E-05 (4.88E-06; 1.11E-04) | **0.03** |
| rs700059 | *STRBP* | G | 3.93 (-4.47; 12.33) | 0.36 | | 17.07 (-3.19; 37.34) | 0.10 | | 7.31 (-35.08; 49.69) | 0.74 | | 5.74E-05 (1.25E-05; 1.02E-04) | **0.01** |
| rs10830963 | *MTNR1B* | G | 6.81 (-0.61; 14.23) | 0.07 | | 10.48 (-7.58; 28.54) | 0.25 | | -0.58 (-38.41; 37.24) | 0.98 | | 3.32E-05 (2.66E-07; 6.60E-05) | **0.05** |
| rs138715366 | *YKT6-GCK* | C | -6.81 (-14.23; 0.61) | 0.07 | | -10.48 (-28.54; 7.58) | 0.25 | | 0.58 (-37.24; 38.41) | 0.98 | | 2.04E-04 (6.12E-06; 4.04E-04) | **0.04** |
| rs2150052 | *LPAR1* | T | -3.92 (-12.11; 4.28) | 0.35 | | -8.39 (-28.23; 11.44) | 0.41 | | -10.54 (-51.84; 30.77) | 0.62 | | 3.14E-05 (9.96E-07; 6.16E-05) | **0.04** |
| rs3753639 | *ZBTB7B* | C | 3.11 (-4.56; 10.77) | 0.43 | | -1.75 (-20.18; 16.68) | 0.85 | | 15.94 (-22.44; 54.33) | 0.42 | | 2.32E-05 (-1.40E-05; 6.06E-05) | 0.22 |
| rs61830764 | *DTL* | A | 16.04 (3.62; 28.45) | **0.01** | | -9.44 (-40.41; 21.53) | 0.55 | | 68.98 (4.58; 133.39) | **0.04** | | 3.34E-06 (-2.88E-05; 3.56E-05) | 0.84 |
| rs61862780 | *HHEX-IDE* | T | 8.89 (1.53; 16.25) | **0.02** | | 3.91 (-14.05; 21.87) | 0.67 | | 1.50 (-36.05; 39.05) | 0.94 | | -6.02E-06 (-3.76E-05; 2.56E-05) | 0.71 |
| rs6989280 | *TRIB1* | G | -6.04 (-14.50; 2.42) | 0.16 | | 20.84 (0.42; 41.27) | **0.05** | | -19.02 (-61.69; 23.66) | 0.38 | | -1.77E-05 (-5.22E-05; 1.68E-05) | 0.31 |
| rs28510415 | *PTCH1* | G | 1.62 (-8.60; 11.85) | 0.76 | | 43.12 (18.33; 67.92) | **<0.01** | | 26.27 (-26.57; 79.10) | 0.33 | | -3.36E-06 (-5.08E-05; 4.40E-05) | 0.89 |
| rs62466330 | *MLXIPL* | C | -4.59 (-11.90; 2.72) | 0.22 | | 16.52 (-1.06; 34.10) | 0.07 | | 39.33 (2.50; 76.16) | **0.04** | | -4.10E-05 (-1.01E-04; 1.92E-05) | 0.18 |
| rs2324499 | *LINC00332* | G | -2.93 (-10.43; 4.57) | 0.44 | | 6.58 (-11.62; 24.77) | 0.48 | | -50.82 (-88.56; -13.08) | **0.01** | | 2.96E-05 (-2.66E-06; 6.18E-05) | 0.07 |
| rs2229742 | *NRIP1* | G | 3.52 (-5.05; 12.10) | 0.42 | | 16.23 (-4.64; 37.11) | 0.13 | | -7.66 (-51.30; 35.97) | 0.73 | | 3.78E-05 (-3.80E-06; 7.96E-05) | 0.08 |
| rs2473248 | *WNT4-ZBTB40* | C | -3.95 (-13.35; 5.46) | 0.41 | | 5.90 (-16.91; 28.70) | 0.61 | | 14.01 (-33.73; 61.74) | 0.56 | | 3.38E-05 (-9.74E-06; 7.72E-05) | 0.13 |
| rs6537307 | *HHIP* | G | 4.27 (-3.78; 12.31) | 0.30 | | 5.44 (-13.98; 24.86) | 0.58 | | 43.00 (2.47; 83.53) | **0.04** | | 2.72E-05 (-3.08E-06; 5.76E-05) | 0.08 |
| rs2854355 | *RB1* | G | -6.79 (-15.58; 2.00) | 0.13 | | 5.94 (-15.47; 27.35) | 0.59 | | -19.07 (-63.43; 25.29) | 0.40 | | 1.35E-05 (-2.10E-05; 4.80E-05) | 0.44 |
| rs11719201 | *ADCY5* | T | 6.88 (-0.99; 14.74) | 0.09 | | -2.56 (-21.67; 16.54) | 0.79 | | 3.70 (-36.01; 43.42) | 0.85 | | -2.14E-05 (-5.26E-05; 9.84E-06) | 0.18 |
| rs10935733 | *CPA3* | T | -0.85 (-10.44; 8.74) | 0.86 | | -13.48 (-36.83; 9.87) | 0.26 | | 40.60 (-7.95; 89.16) | 0.10 | | 2.14E-05 (-9.84E-06; 5.26E-05) | 0.18 |
| rs13266210 | *ANK1-NKX6.3* | A | 1.25 (-6.86; 9.36) | 0.76 | | 1.96 (-17.59; 21.50) | 0.84 | | -19.25 (-60.17; 21.66) | 0.36 | | 1.21E-05 (-2.74E-05; 5.16E-05) | 0.55 |
| rs798489 | *GNA12* | C | -18.09 (-66.81; 30.62) | 0.47 | | 105.63 (-8.61; 219.87) | 0.07 | | 126.16 (-114.72; 367.04) | 0.30 | | -2.16E-05 (-5.62E-05; 1.29E-05) | 0.22 |
| rs1819436 | *RNF219-AS1* | C | -10.13 (-24.74; 4.47) | 0.17 | | -12.97 (-48.20; 22.25) | 0.47 | | -8.30 (-81.30; 64.71) | 0.82 | | 1.71E-05 (-3.54E-05; 6.94E-05) | 0.52 |
| rs1351394 | *HMGA2* | T | -2.66 (-10.47; 5.15) | 0.50 | | -12.22 (-31.10; 6.66) | 0.20 | | 29.51 (-10.03; 69.05) | 0.14 | | 1.80E-05 (-1.32E-05; 4.92E-05) | 0.26 |
| rs2242116 | *PTH1R* | A | 0.83 (-7.45; 9.10) | 0.84 | | -16.65 (-36.78; 3.47) | 0.10 | | -3.45 (-45.26; 38.36) | 0.87 | | -1.82E-05 (-4.96E-05; 1.31E-05) | 0.26 |
| rs6959887 | *TBX20* | A | 0.60 (-6.88; 8.08) | 0.88 | | -10.72 (-28.85; 7.41) | 0.25 | | 34.37 (-3.13; 71.87) | 0.07 | | -1.34E-06 (-3.40E-05; 3.14E-05) | 0.94 |
| rs12823128 | *ITPR2* | T | 0.70 (-8.88; 10.27) | 0.89 | | -6.09 (-29.01; 16.82) | 0.60 | | 13.73 (-33.79; 61.25) | 0.57 | | 1.07E-05 (-2.10E-05; 4.24E-05) | 0.51 |
| rs854037 | *5q11.2* | A | 0.35 (-7.92; 8.61) | 0.93 | | -12.44 (-32.58; 7.70) | 0.23 | | -23.93 (-65.76; 17.90) | 0.26 | | 1.35E-05 (-2.60E-05; 5.32E-05) | 0.50 |
| rs1011939 | *GPR139* | G | 2.27 (-5.00; 9.54) | 0.54 | | 13.82 (-3.72; 31.36) | 0.12 | | 24.96 (-11.85; 61.77) | 0.18 | | 1.02E-05 (-2.36E-05; 4.40E-05) | 0.55 |
| rs134594 | *KREMEN1* | C | 7.90 (-3.44; 19.23) | 0.17 | | -15.53 (-42.84; 11.78) | 0.26 | | -1.91 (-59.05; 55.23) | 0.95 | | -5.66E-06 (-3.76E-05; 2.62E-05) | 0.73 |
| rs1101081 | *ESR1* | C | 7.99 (-2.91; 18.89) | 0.15 | | 23.36 (-2.70; 49.43) | 0.08 | | 30.07 (-25.06; 85.19) | 0.28 | | 2.98E-05 (-3.68E-06; 6.34E-05) | 0.08 |
| rs7729301 | *EBF1* | A | 2.97 (-4.39; 10.34) | 0.43 | | -8.72 (-26.55; 9.12) | 0.34 | | 12.60 (-24.69; 49.89) | 0.51 | | 1.53E-05 (-2.08E-05; 5.14E-05) | 0.41 |
| rs62240962 | *SREBF2* | C | -0.74 (-8.37; 6.89) | 0.85 | | -5.45 (-23.74; 12.84) | 0.56 | | 5.12 (-33.03; 43.27) | 0.79 | | 7.64E-06 (-4.80E-05; 6.32E-05) | 0.79 |
| rs6016377 | *MAFB* | T | -0.94 (-13.28; 11.41) | 0.88 | | 8.47 (-21.35; 38.30) | 0.58 | | -8.49 (-69.88; 52.91) | 0.79 | | 9.50E-06 (-2.26E-05; 4.16E-05) | 0.56 |
| rs113086489 | *CLDN7* | T | -0.58 (-8.42; 7.26) | 0.88 | | 15.56 (-3.59; 34.72) | 0.11 | | 26.71 (-13.14; 66.57) | 0.19 | | 1.65E-05 (-1.41E-05; 4.72E-05) | 0.29 |
| rs10402712 | *PEPD* | A | -0.36 (-9.59; 8.87) | 0.94 | | -4.08 (-26.32; 18.17) | 0.72 | | 20.82 (-25.64; 67.28) | 0.38 | | -1.93E-06 (-3.62E-05; 3.22E-05) | 0.91 |
| rs11055034 | *APOLD1* | C | -4.02 (-11.64; 3.60) | 0.30 | | 1.82 (-16.59; 20.23) | 0.85 | | 3.87 (-34.66; 42.39) | 0.84 | | 3.88E-06 (-3.46E-05; 4.24E-05) | 0.84 |
| rs74233809 | *NT5C2* | C | 12.58 (-1.72; 26.89) | 0.08 | | 26.35 (-8.84; 61.53) | 0.14 | | 28.15 (-45.28; 101.57) | 0.45 | | -5.36E-06 (-5.54E-05; 4.46E-05) | 0.83 |
| rs6040076 | *JAG1* | C | 1.55 (-10.80; 13.90) | 0.80 | | 17.37 (-12.76; 47.50) | 0.26 | | -41.48 (-104.95; 21.99) | 0.20 | | -6.08E-07 (-3.30E-05; 3.18E-05) | 0.97 |
| rs1415701 | *L3MBTL3* | G | 5.66 (-2.10; 13.41) | 0.15 | | -4.86 (-23.62; 13.89) | 0.61 | | 36.53 (-2.66; 75.73) | 0.07 | | -9.62E-06 (-4.64E-05; 2.72E-05) | 0.61 |
| rs9379832 | *HIST1H2BE* | A | 0.08 (-8.18; 8.34) | 0.99 | | 11.97 (-8.03; 31.98) | 0.24 | | -3.87 (-45.83; 38.10) | 0.86 | | -7.22E-06 (-4.10E-05; 2.66E-05) | 0.68 |
| rs28530618 | *C20orf203* | A | 2.82 (-5.20; 10.84) | 0.49 | | -9.80 (-29.25; 9.65) | 0.32 | | 19.28 (-21.45; 60.01) | 0.35 | | -4.44E-06 (-3.54E-05; 2.66E-05) | 0.78 |
| rs12543725 | *SLC45A4* | G | -3.50 (-10.88; 3.88) | 0.35 | | 8.50 (-9.24; 26.23) | 0.35 | | 34.98 (-1.77; 71.74) | 0.06 | | 1.24E-05 (-1.88E-05; 4.36E-05) | 0.44 |
| rs2421016 | *PLEKHA1* | T | 7.73 (-28.39; 43.86) | 0.67 | | 66.69 (-20.02; 153.40) | 0.13 | | -123.55 (-299.84; 52.74) | 0.17 | | -1.56E-06 (-3.24E-05; 2.92E-05) | 0.92 |
| rs35261542 | *CDKAL1* | C | 2.24 (-5.87; 10.35) | 0.59 | | 8.55 (-11.16; 28.26) | 0.39 | | -33.04 (-74.30; 8.21) | 0.12 | | 7.54E-06 (-2.54E-05; 4.04E-05) | 0.65 |
| rs7742369 | *HMGA1* | G | 0.22 (-7.07; 7.52) | 0.95 | | -13.76 (-31.56; 4.05) | 0.13 | | 21.33 (-16.15; 58.81) | 0.26 | | 5.12E-06 (-3.52E-05; 4.54E-05) | 0.80 |
| rs7076938 | *ADRB1* | T | 0.25 (-7.41; 7.92) | 0.95 | | 4.09 (-14.56; 22.73) | 0.67 | | 7.03 (-31.86; 45.91) | 0.72 | | -8.08E-07 (-3.62E-05; 3.46E-05) | 0.96 |
| rs13322435 | *CCNL1-LEKR1* | A | -0.86 (-8.68; 6.97) | 0.83 | | -7.16 (-26.07; 11.74) | 0.46 | | 18.58 (-20.74; 57.91) | 0.35 | | 8.84E-06 (-2.28E-05; 4.06E-05) | 0.59 |
| rs925098 | *LCORL* | G | 8.87 (-1.13; 18.88) | 0.08 | | 10.77 (-13.30; 34.84) | 0.38 | | 42.40 (-7.92; 92.73) | 0.10 | | -9.36E-06 (-4.36E-05; 2.48E-05) | 0.59 |
| rs72480273 | *FCGR2B* | C | 0.56 (-7.08; 8.20) | 0.89 | | -8.90 (-27.37; 9.58) | 0.34 | | -3.27 (-41.84; 35.30) | 0.87 | | 5.90E-07 (-4.18E-05; 4.30E-05) | 0.98 |
| rs144843919 | *SUZ12P1-CRLF3* | G | 7.49 (-5.64; 20.62) | 0.26 | | 4.42 (-27.79; 36.62) | 0.79 | | -28.89 (-95.69; 37.92) | 0.40 | | -1.57E-05 (-1.62E-04; 1.31E-04) | 0.83 |
| rs11763171  *proxy for rs11765649 (r^2^= 0.90) | *IGF2BP3* | G | -6.49 (-15.3; 2.38) | 0.11 | | 1.98 (-19.3; 23.2) | 0.86 | | 6.44 (-38.0; 50.2) | 0.78 | | -1.57E-04 (-5.39E-04; 2.65E-04) | 0.42 |
| rs8032315  *proxy for rs12906125  (r^2^= 0.98) | *FURIN* | T | -0.90 (-9.03; 7.21) | 0.54 | | -9.36 (-29.1; 10.4) | 0.22 | | 33.7 (-7.6;75.0) | 0.17 | | -4.26E-05 (-3.94E-04; 3.09E-04) | 0.69 |
| rs8066574  *proxy for rs12942207  (r^2^= 1) | *LOC102724532* | G | 3.14 (-4.98; 11.3) | 0.31 | | 8.18 (-11.6; 28.0) | 0.61 | | -9.85 (-51.2;31.5) | 0.54 | | 1.24E-04 (-5.38E-04; 2.25E-04) | 0.49 |
| rs8034564  *proxy for rs7402982  (r^2^= 0.94) | *IGF1R* | G | -2.27 (-9.89; 5.36) | 0.78 | | -0.126 (-18.6; 18.4) | 0.98 | | -4.63 (-43.3; 34.1) | 0.85 | | 4.43E-06 (-3.26E-04; 3.35E-04) | 0.98 |
| rs139675912  *proxy for rs7575873  (r^2^= 1) | *ATAD2B* | C | 0.07 (-11.0; 11.20) | 0.79 | | -14.3 (-40.9; 12.2) | 0.23 | | -14.9 (-71.3; 40.5) | 0.70 | | -1.05E-04 (-5.85E-04; 3.74E-04) | 0.67 |
| rs4836833  *proxy for rs7847628  (r^2^= 1) | *PHF19* | G | 3.18 (-4.65; 11.0) | 0.61 | | 8.33 (-10.8; 27.4) | 0.55 | | -16.7 (-56.7;23.3) | 0.44 | | 1.03E-04 (-3.38E-04; 4.43E-04) | 0.55 |

EA: Effect allele. p values and beta estimates were (for week 25, 32 and birth) calculated by multiple linear regression models assessing the relationship between fetal weight (g) at week 25/32/birth and the variant adjusted for gestational age (GA), fetal weight and gestational age at the previous ultrasound scan (week 20, 25 or 32), fetal gender and maternal pre-pregnancy BMI. Overall intrauterine growth (including birth weight) was assessed by linear mixed models adjusted for gestational age (GA), GA^2^, and for interactions between GA and GA^2^ with fetal gender and maternal BMI.
